# Coding complete genome sequence of the variant of eggplant mild leaf mottle virus from Najaf, Iraq

**DOI:** 10.1128/mra.01341-25

**Published:** 2026-02-03

**Authors:** Malak Faeq, Osamah Alisawi

**Affiliations:** 1 Plant Protection Department, Faculty of Agriculture, University of Kufa 125666, Najaf, Iraq; Katholieke Universiteit Leuven, Leuven, Belgium

**Keywords:** RNA seq, bioinformatics, eggplant genome, *Eggplant mild leaf mottle virus*

## Abstract

Here, we report the coding complete genome sequence of a variant of Eggplant mild leaf mottle virus in eggplant using RNA seq. The phylogeny revealed that the isolate clustered with strains from India, Iraq, Israel, and Canada.

## ANNOUNCEMENT

**Fig 1:**
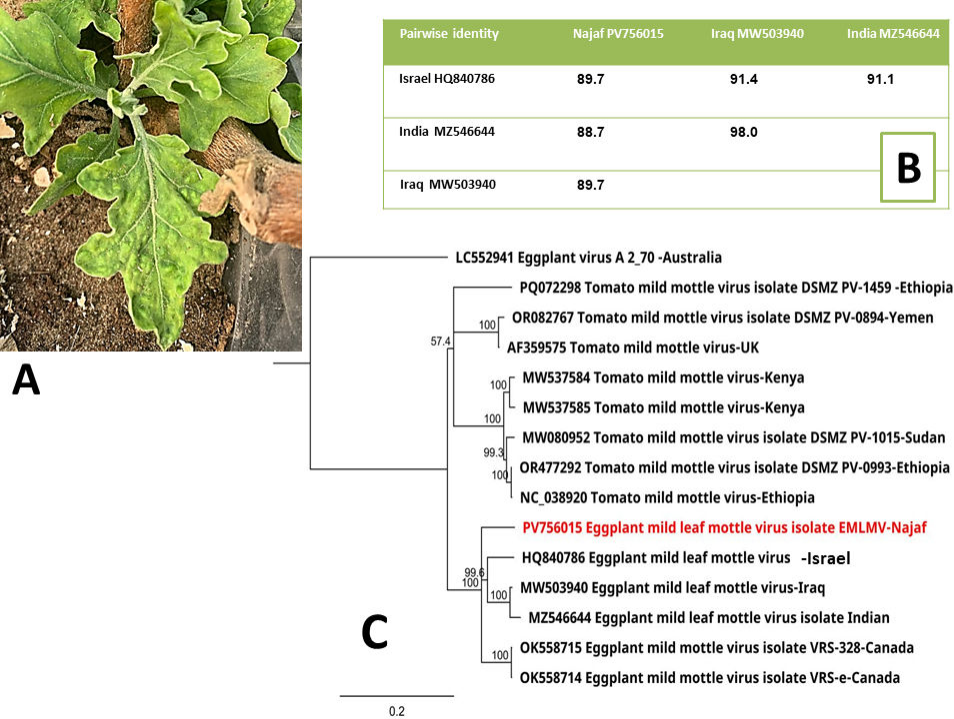
Eggplant leaves displayed mosaic and curling symptoms (**A**), pairwise nucleotide similarities between the Najaf isolate and related isolates from Iraq, Israel, and India (**B**), and ClustalW was used to align the full-genome nucleotide sequences. The neighbor-joining method was used in combination with the best substitution model (Hasegawa-Kishino-Yano) to build the tree in Geneious Tree Builder, applying 1,000 bootstraps, the strain from this study is indicated in red (**C**). Bar scale was 0.2.

Eggplant has been identified as a host for various viruses (1). Recently, the Jordan and Arava Valleys were affected by a novel disease that causes mild leaf mottling and fruit distortion on eggplants (2). The symptoms were caused by a viral agent named Eggplant mild leaf mottle virus (EMLMV) that belongs to the *Potyviridae* family, specifically the atypical genus *Ipomovirus*, that is transmitted by a mechanical inoculation and whitefly. The virus genome is composed of a linear ssRNA molecule of 9,280 nucleotides and encodes a large open reading frame composed of 3,011 amino acids (3). Furthermore, this virus has a filamentous shape (720 nm long) and can present with cytoplasmic pinwheels and crystalline structures, similar to Tomato mild mottle virus. Interestingly, whiteflies play a major role in the virus transmission, which is uncharacteristic for *Potyviridae* members (2). On 2 April 2025, the leaves of diseased eggplant from the Najaf Province in Iraq were collected (Fig. 1A), chopped into 0.5×0.5 cm squares, submerged in five times the volume of RNA later solution. The RNA extraction and sequencing were performed by JS Link (Seoul, Korea). The RNeasy plant micro kit (Qiagen, Hilden, Germany) was used to extract total RNA in compliance with the company’s instructions. The company employed the entire RNA library prep kit from TruSeq to get the Illumina library ready for sequencing. Whole genome sequencing was performed on RNA with the WGS application (PCR Free550: Platform: NovaseqX; Application: WTS/mRNA). Trimmomatic-0.39 (4) was used to trim 77,453,794 raw reads, yielding 77,376,482 high-quality, clean, paired-end reads with 101 bases. The Geneious Prime 2025 software’s Geneious RNA mapper (Sensitivity: Medium-Low Sensitivity) (5) was used to map the paired-end reads to the expected eggplant virus genomes. The reference genome of EMLMV (MW503940) (6) was covered for 100% with 4,402,240 reads, and the consensus sequence was extracted. Pairwise nucleotide alignments were performed between the examined virus isolate and closely related isolates from GenBank (HQ840786, MW503940, and MZ546644), and their nt similarity ranged from 88.7 to 89.7% (Fig. 1B). Geneious Prime 2025.1.3’s Open Reading Frame Finder (5) and BLASTx version 5 (7) were utilized for annotation. Alignments were generated using ClustalW, and a tree was built using the Geneious tree builder. Unless otherwise noted, all tools were run with the default settings. The entire 9,282 nt virus genome sequence was deposited to GenBank with accession number PV756015, and the genome sequence encoded 3,011 aa polyprotein. The coverage depth was 116,285× and GC content was 43.8%. Phylogenetic analyses clustered the Najaf isolate together with strains from Israel, Iraq, India, and Canada (Fig. 1C). The study demonstrated the importance of diagnosing newly emerging variants of such viruses for plant health management and biosecurity.

## Data Availability

The coding complete genome sequence of Eggplant mild leaf mottle virus has been deposited in GenBank under accession number PV756015. The raw reads were deposited in the Sequence Read Archive under SRR35013253.
